# Molecular Mechanisms of Action of FSH

**DOI:** 10.3389/fendo.2019.00305

**Published:** 2019-05-14

**Authors:** Livio Casarini, Pascale Crépieux

**Affiliations:** ^1^Unit of Endocrinology, Department Biomedical, Metabolic and Neural Sciences, University of Modena and Reggio Emilia, Modena, Italy; ^2^Center for Genomic Research, University of Modena and Reggio Emilia, Modena, Italy; ^3^PRC, UMR INRA0085, CNRS 7247, Centre INRA Val de Loire, Nouzilly, France

**Keywords:** FSH, FSHR, signaling, PKA, arrestin

## Abstract

The glycoprotein follicle-stimulating hormone (FSH) acts on gonadal target cells, hence regulating gametogenesis. The transduction of the hormone-induced signal is mediated by the FSH-specific G protein-coupled receptor (FSHR), of which the action relies on the interaction with a number of intracellular effectors. The stimulatory Gαs protein is a long-time known transducer of FSH signaling, mainly leading to intracellular cAMP increase and protein kinase A (PKA) activation, the latter acting as a master regulator of cell metabolism and sex steroid production. While *in vivo* data clearly demonstrate the relevance of PKA activation in mediating gametogenesis by triggering proliferative signals, some *in vitro* data suggest that pro-apoptotic pathways may be awakened as a “dark side” of cAMP/PKA-dependent steroidogenesis, in certain conditions. P38 mitogen-activated protein kinases (MAPK) are players of death signals in steroidogenic cells, involving downstream p53 and caspases. Although it could be hypothesized that pro-apoptotic signals, if relevant, may be required for regulating *atresia* of *non*-dominant ovarian follicles, they should be transient and counterbalanced by mitogenic signals upon FSHR interaction with opposing transducers, such as Gαi proteins and β-arrestins. These molecules modulate the steroidogenic pathway *via* extracellular-regulated kinases (ERK1/2), phosphatidylinositol-4,5-bisphosphate 3-kinases (PI3K)/protein kinase B (AKT), calcium signaling and other intracellular signaling effectors, resulting in a complex and dynamic signaling network characterizing sex- and stage-specific gamete maturation. Even if the FSH-mediated signaling network is not yet entirely deciphered, its full comprehension is of high physiological and clinical relevance due to the crucial role covered by the hormone in regulating human development and reproduction.

## Introduction

Follicle-stimulating hormone (FSH) is a glycoprotein playing a central role in mammalian reproduction and development. In the ovary, FSH regulates folliculogenesis, oocyte selection, and the synthesis of sex steroid hormones, thus preparing the reproductive tract for fertilization, implantation, and pregnancy (1). In the male, this gonadotropin mediates testicular development and spermatogenesis (2). The hormone is secreted by the gonadotrope cells of the pituitary, upon pulsatile regulation by the hypothalamic gonadotropin-releasing hormone (GnRH) (3), and acts on the surface of target cells located in the gonads of both males and females, where hormone-induced cell proliferation- and apoptosis-linked signals are triggered. FSH displays an α subunit, common to other gonadotropins and to thyrotropin, and a β subunit specifically binding to its G protein-coupled receptor (GPCR), namely FSHR (4). *In silico* and crystallographic structural analyzes found also interaction between the α subunit and FSHR, demonstrating that receptor binding is not exclusive of the β subunit (5). Hormone binding implies conformational changes of the receptor (6) that transduce the signal *via* direct protein interactions at the plasma membrane, resulting in a cascade of biochemical reactions that constitute an intertwined complex signaling network (7). In this review, signaling pathways activated in gonadal cells upon FSH binding to its membrane receptor are discussed in detail, providing a comprehensive view on the downstream life and death signals regulating reproductive functions.

## FSHR Interaction With Membrane Receptors

The FSHR has been shown to functionally and/or physically interact with other membrane receptors (8, 9), hence intensifying the diversity of FSH action (10). For example, the FSHR may exist as a unit of di/trimeric homomers (5). Interestingly, heterodimerization of the FSHR with the luteinizing hormone (LH) receptor (LHCGR) (11) may play a key role in regulating the ovarian growth and selection (12), by virtue of the physical interaction between these two receptors. Interestingly, intracellular signals delivered by LH at the LHCGR may be modulated by the presence of FSHR on the cell surface, and *vice versa*, through the formation of receptor heteromers. For example, unliganded co-expressed FSHR amplifies Gαq-mediated signaling initiated at the LHCGR (13), whereas the LHCGR may inhibit FSHR-dependent cAMP production (11). In addition, other classes of receptors, such as tyrosine kinase receptors, may also contribute to the modulation of FSHR activity. The insulin-like growth factor-1 receptor (IGF-1R) is one of those, as it appears necessary for FSH-induced granulosa cell differentiation *via* a signaling cascade involving the thymoma viral oncogene homolog 3 (AKT3) (14). Similarly, action of the epidermal growth factor receptor (EGFR) during granulosa cell differentiation is required for activation of ERK1/2 (15). Interestingly, the interaction between FSHR and EGFR signaling networks was analyzed using an automated, logic-based approach, suggesting that the ERK1/2-pathway may be activated by EGFR-dependent signals *via* p38 mitogen-activated protein kinases (MAPK) (16). Moreover, this study confirmed that EGFR is trans-activated through FSHR-mediated pathways involving the proto-oncogene tyrosine-protein kinase *SRC*. On the other hand, EGFR signaling network overlaps, at least in part, that of FSHR, contributing to modulation of the ERK1/2, the phosphatidylinositol-4,5-bisphosphate 3-kinases (PI3K)/protein kinase B (AKT), and the Janus kinase (JAK)/signal transducer and activator of transcription protein (STAT) pathways (16).

## Intracellular FSHR Signal Transducing Partners

Typically, G proteins are directly activated by the FSHR, by splitting of the βγ dimer from the α subunit (17), that act as regulators of intracellular enzymes, such as G protein-coupled receptor kinases (GRKs), or adenylyl cyclase, respectively, among many others (18). Moreover, βγ dimer was demonstrated to be able of modulating intracellular signaling cascades (19, 20).

G protein activation is followed by FSHR phosphorylation at the intracellular level, operated by GRKs and resulting in receptor association with β-arrestins (21, 22). β-arrestins are scaffold proteins (23) that mediate GPCR desensitization, recycling, and G protein-independent signaling (24). Another direct FSHR-interacting partner is adaptor protein, phosphotyrosine interacting with PH domain and leucine zipper 1 (APPL1), that is linked to the activation of the PI3K/AKT anti-apoptotic pathway and calcium ion mobilization (25). By these means, APPL1 might regulate the selection of the dominant follicle by mediating the anti-apoptotic effects exerted by FSH *via* inhibitory phosphorylation of forkhead homolog in rhabdomyosarcoma (FOXO1a) (26). Interestingly, APPL1 is involved in cAMP signaling exerted by GPCR activity in very early endosomal compartments, hence contributing to the spatial encoding of intracellular signaling, as shown for the LHR (27). Similarly, GAIP-interacting protein C terminus (GIPC), a PDZ protein, redirects the FSHR to pre-early endosomes, hence promoting sustained, intracellular MAPK (28). Another protein directly interacting with FSHR is the 14-3-3τ adapter protein (29), which may contact the canonical G protein-receptor interaction site located at the intracellular level and mediates the activation of the AKT-pathway (30).

**Figure 1:**
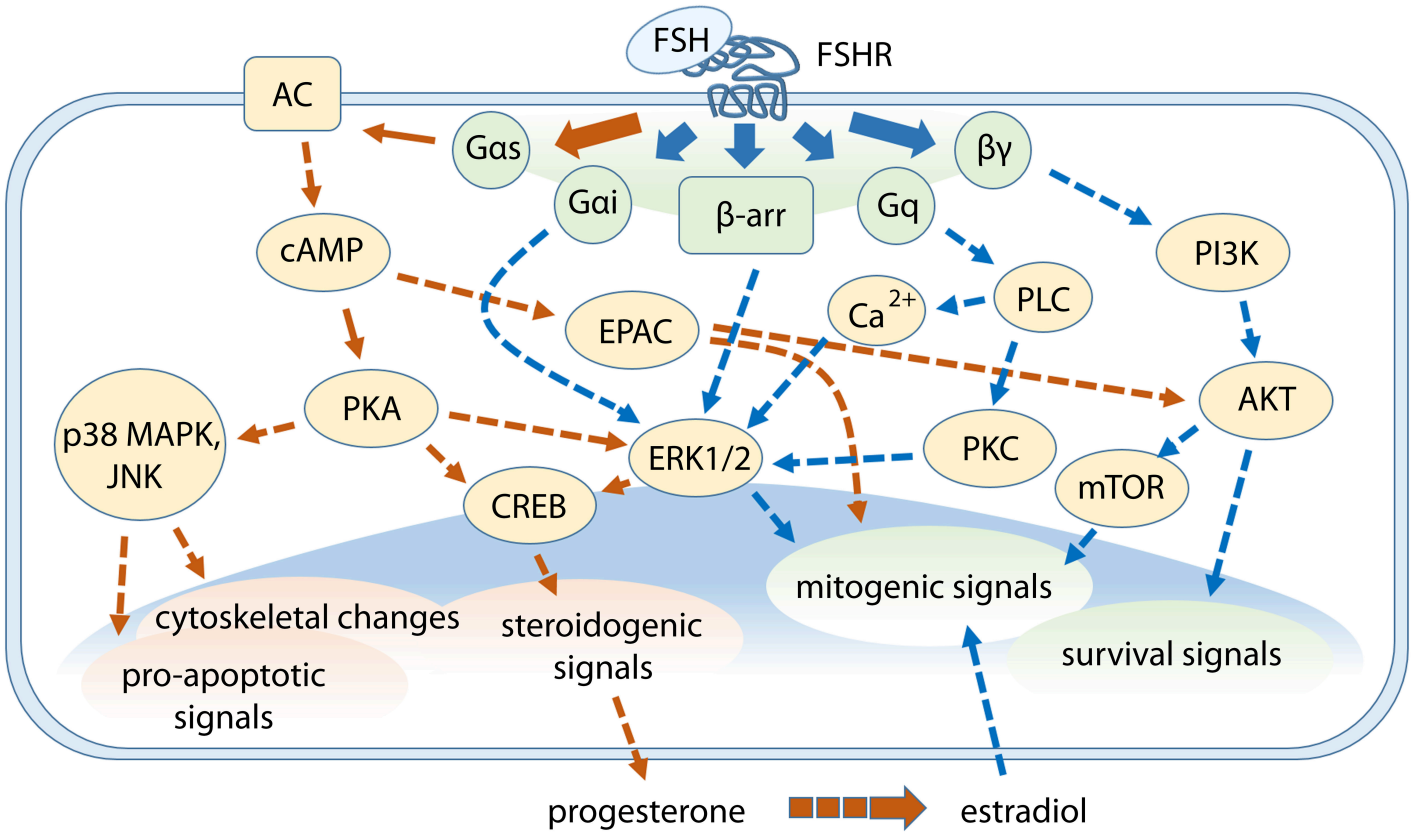
Cross-talk between FSH-dependent steroidogenic, life, and death signals in granulosa cells. G protein subunits and β-arrestins mediate the activation of multiple signaling pathways modulating different events downstream. Gαs protein/cAMP-related signaling are represented by orange arrows while signaling cascades depending on other FSHR intracellular interactors are indicated by blue arrows. Steroidogenic events are mainly mediated through cAMP/PKA-pathway, which is linked to p38 MAPK signaling, while ERK1/2 and AKT are key players for mitogenic and survival signals activation. Some pathways were omitted.

In the gonads, FSH-mediated signaling results in the transcription of target genes, which include *LHCGR* and other genes encoding membrane receptors, protein kinases, growth factors, enzymes regulating steroid synthesis, genes involved in the regulation of cell cycle, proliferation and differentiation, apoptosis, and circadian rhythm (31–33). Despite the wide diversity of FSH target genes, effects of gonadal stimulation by the hormone was defined as both proliferative and anti-apoptotic due to the positive impact on gametogenesis (34, 35) and on growth of certain cancer cells (36). Nevertheless, pro-apoptotic functions emerged as a condition related to FSH-mediated steroid production (37, 38). In this review, molecular mechanisms of FSH action and their relationships with downstream steroidogenic, life, and death signals regulating reproduction (Figure 1) are discussed.

## Activation of the cAMP/PKA Steroidogenic Pathway

While FSH is mainly known to support the maturation of gametes *via* Sertoli cell nurturing functions in the male, the hormone has steroidogenic activity in ovarian granulosa cells (4). This action is exerted *via* the protein kinase A (PKA) pathway, whose activation depends on ATP conversion into the second messenger cAMP by adenylyl cyclases, primary targets of the Gαs protein subunit. The interaction between cAMP and PKA was described several decades ago (39). Intracellular cAMP increase is under the negative control of phosphodiesterase (PDE) enzymes, which metabolize the second messenger into 5′AMP (40). As mentioned above, cAMP signaling is spatially and temporally compartmentalized within the cell (41). Versatility in cAMP-dependent signaling depends on the expression of factors such as the isoform of adenylyl cyclase (42), PDE (43), β-arrestins (44), and A kinase anchoring proteins (AKAP) (45) that target the subcellular distribution of PKA.

**Figure 2:**
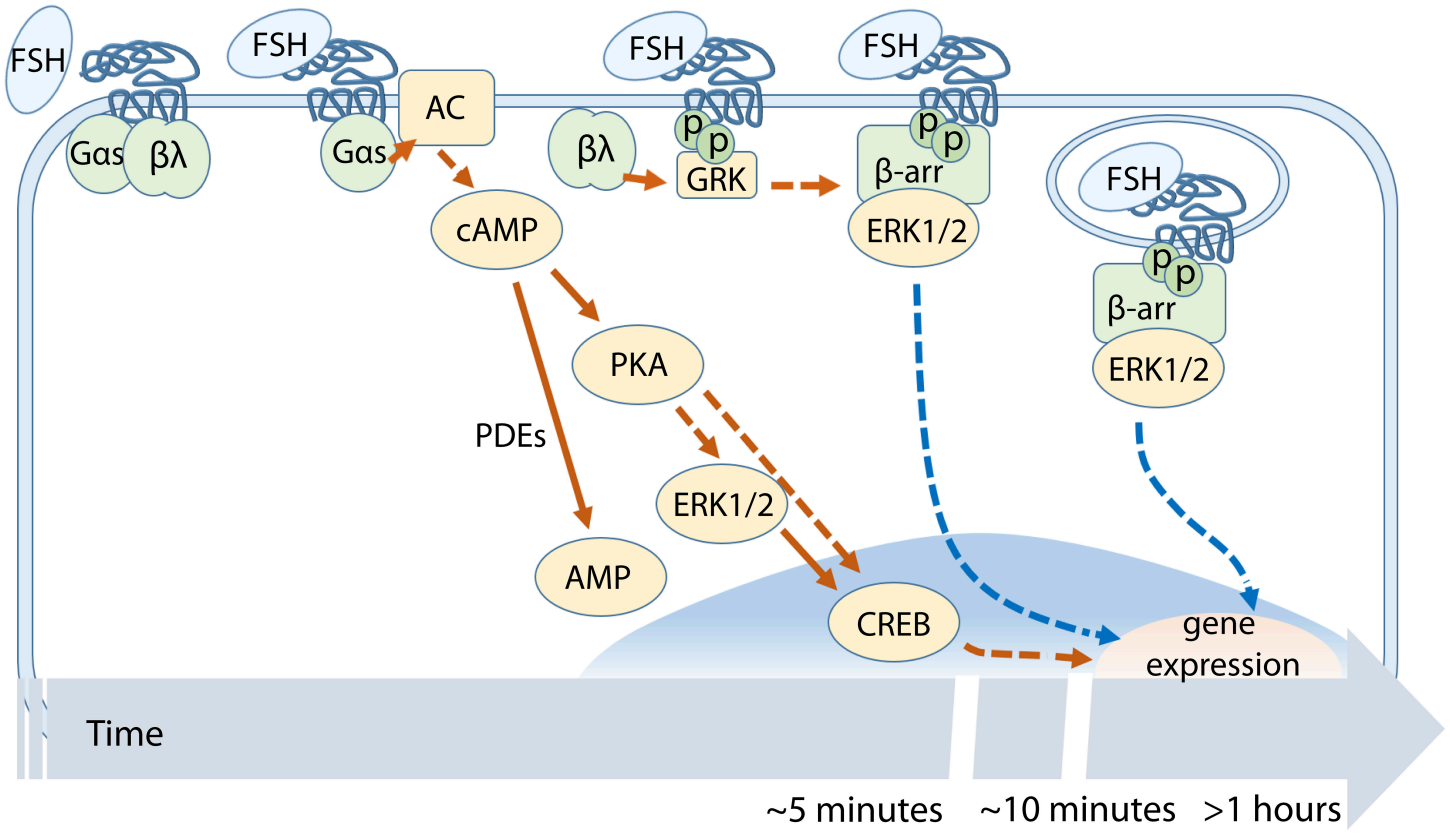
Temporal succession of FSH-dependent events across the cAMP/PKA-pathway. cAMP-related signaling involves PKA, ERK1/2, and CREB activation. FSHR phosphorylation by GRKs occurs before β-arrestin recruitment and subsequent receptor internalization.

In Sertoli cells, cAMP binding to PKA results in the release of PKA catalytic subunits (46) and indirectly mediates the phosphorylation of the extracellular signal-regulated kinase 1/2 (ERK1/2) MAPK, in order to promote cell proliferation (47). In granulosa cells, the mechanism whereby ERK is activated likely consists in the removal of a tonic inhibition exerted by a phosphotyrosine phosphatase on MEK1 (48), recently identified as DUSP6 (49). An alternative mechanism consists in the activation of ERK1/2 by β-arrestins, with a different kinetics than G proteins (Figure 2), since it is delayed and sustained (50). It was demonstrated that pERK1/2 is involved in both cAMP-dependent (51) and -independent (52) steroidogenesis. In the first case, depletion of ERK1/2 phosphorylation by specific MEK inhibition resulted in attenuated early (10–15 min) phosphorylation of the cAMP response element-binding protein (CREB) (51), a nuclear transcription factor up-regulating steroidogenic enzymes in gonadal cells (53). In this case, pERK1/2 inhibition negatively impacts on progesterone synthesis, indicating that cAMP-dependent ERK1/2 phosphorylation plays a stimulatory role in the rapidly delivered FSH-dependent steroidogenic signal. Interestingly, molecular mechanisms regulating steroidogenic stimuli in the Leydig cell may be different to those occurring in FSH-responsive cells. In Leydig cells, steroid hormones may be produced *via* ERK1/2- and CREB-dependent signaling in the absence of cAMP recruitment, *via* an EGFR-regulated mechanism (52). In granulosa cells, selective blockade of MAPK activation results in the inhibition of FSH-dependent StAR and progesterone synthesis while androgens to estrogen conversion by the enzyme aromatase is enhanced (54), demonstrating a differential regulation of FSH-induced sex steroid synthesis in target cells. Similar results were found by treating theca cells with LH, that induced differential, ERK1/2-dependent regulation of progesterone and androgen production (55). However, the role of ERK1/2 in mediating steroidogenesis is a still debated matter, since it was reported to be inhibitory (56) while other studies demonstrated the positive impact of the MAPK activation on the synthesis of sex steroids (57).

## Roles of cAMP-dependent PKA Activation

Whereas, ERK is an indirect cytosolic target of PKA that can affect CREB phosphorylation (51), the latter may be directly activated upon translocation of PKA catalytic subunit in the nucleus (48), hence inducing the transcription of CREB target genes characterized by cAMP-response elements (CRE) within their promoter region (53). Nuclear PKA was also shown to phosphorylate histone H3, thus mediating FSH mitogenic activity in granulosa cells (58, 59). These interesting observations suggest that PKA could be endowed with a more general role in gene transcription, by promoting chromatin remodeling through histone H3 post-translational modifications. In addition, recent genome-wide experiments have highlighted that FSH-responsive genes contain far less *CRE* than expected in their promoters, that are notably enriched in GATA-binding sites (32).

The wide range of PKA-dependent signaling pathways suggests that the kinase is a master regulator of several FSH-dependent cell functions, especially those related to steroidogenesis and cell differentiation. However, intracellular signaling cascades regulated by PKA do not completely overlap those depending on FSH. For example, FSH induces p38 MAPK activation while PKA *per se* does not (60).

FSH-induced cAMP production does not only lead to activation of PKA but also of the exchange protein directly activated by cAMP (EPAC) activation. EPAC is a relatively newly discovered cAMP target mediating the activation of the small GTPases RAS and RAP and resulting in the regulation of several cell functions, such as mitogen-activated protein kinase activation, cytoskeletal changes, and calcium homeostasis (61). EPAC was suggested to be a modulator of EGFR expression (62) and granulosa cell differentiation (15) in the ovary, as well as AKT phosphorylation in Sertoli cells (63). However, the role of EPAC in the FSH-mediated signaling cascade is not yet completely elucidated.

## Regulation of Proliferative and pro-apoptotic Signals

In gonadal cells, part of the steroidogenic process and the proteasome are compartmentalized into different organelles, avoiding cell collapse before adequate amount of sex steroid hormones are produced (64). This function is likely enabled to limit the number of follicles that can achieve ovulation and to maintain intact the synthesis of sex steroids during the initial steps of apoptosis. These issues reflect the connection between intracellular signaling cascades regulating steroidogenic signals and pro-apoptotic stimuli, whose dominance is stage-specific, depends on several paracrine factors and is regulated *via* a complex intracellular network involving cAMP and activating the pro-apoptotic protein p53 (65). In this context, the link between cAMP/PKA and p38 MAPK activation may provide a molecular mechanism of apoptosis in steroidogenic cells. The role of p38, as well as Jun N-terminal kinase (JNK), is associated to apoptotic events in pre-ovulatory granulosa cells of primates (66), suggesting that these enzymes could be involved in the selection of the dominant follicle. This role would be counteracted by pERK1/2 activation in the dominant follicle (57), confirming the anti-apoptotic and proliferative functions mediated by this MAP kinase. Indeed, ovarian granulosa cell death is associated with reduced ERK1/2 activity, that is linked to phosphorylation of BCL-2 associated agonist of cell death (BAD) protein leading to a loss of its pro-apoptotic activity (67, 68).

## Pro- and anti-apoptotic Pathways are Activated Simultaneously

In steroidogenic cells, apoptosis is preceded by cell rounding, a cAMP-dependent conformational changes involving actin filaments breakdown (69, 70) that can be prevented by selective blockade of PKA, and also depends on p38 MAPK (71). Both PKA and p38 MAPK may be activated by FSH in a dose-dependent manner, resulting in cytoskeletal rearrangements and shape changes. These data suggest that the gonadotropin retains both pro- and anti-apoptotic potential, exerted *vi*a p38 MAPK and ERK1/2, respectively, and this dual action of FSH provides an interesting point of view on gonadotropin functioning. On the one side, the hormone induces the synthesis of steroid hormones *via* the cAMP/PKA-pathway, as a requisite for gamete growth and reproduction (72). However, the steroidogenic signaling cascade is cross-linked to pro-apoptotic signals occurring through p38 MAPK, activated simultaneously and necessary for regulating steroid synthesis (73, 74). This cross-talk was described even in the mouse adrenal Y1 cell line, where p38 MAPK activation negatively impacts on CREB phosphorylation and StAR activity, inhibiting FSH-induced steroid synthesis (75). On the other side, survival signals are provided through the PKA/ERK1/2 signaling package, counterbalancing the pro-apoptotic effect and, to a certain extent, even inhibiting steroidogenesis (56). While further efforts should be performed to fully solve this question, some hints suggest that the FSH-dependent molecular mechanism underlying cell fate may depend on the potency and persistence of cAMP at the intracellular levels. Indeed, proliferative signals could be predominant at relatively low FSHR expression levels (38), due to preferential activation of ERK1/2 signaling through β-arrestins (38, 76). Relatively high and persistent intracellular cAMP levels due to β-arrestin depletion or FSHR over-expression result in caspase 3 cleavage and apoptosis (38) and this mechanism could contribute to regulating the selection of the dominant ovarian follicles (12). In granulosa cells, *FSHR* over-expression is linked to upregulation of pro-apoptotic genes and increased cell death, compared to cells expressing relatively low *FSHR* levels (77). Thus, it is possible that proliferative signals exerted *via* ERK1/2-pathway could be not sufficient to counteract the pro-apoptotic stimulus during the early/mid-antral follicular phase, when FSHR expression achieves maximal levels (78). In the ovary, this situation should be dynamic and transient, as well as the *FSHR* over-expression (78), follicle-specific and stage-dependent, in order to coordinate the maturation of one single follicle achieving ovulation while the others become *atretic*. This regulatory mechanism may be juxtaposed to what was previously described in Sertoli cell, that is assumed to be the male counterpart of granulosa cell. In 5-day rat Sertoli cells, the ERK1/2-pathway is stimulated by FSH upon dual coupling of FSHR to both stimulatory Gαs and inhibitory Gαi proteins, resulting in cyclin D1 activation and cell proliferation (47). As cells proceed throughout the differentiation program, FSH treatment is linked to consistent ERK1/2 inhibition and decreased cell proliferation, while gradually stabilizing PTEN (79). Thus, the ERK1/2 signaling pathway is a key regulator of FSH-induced life and death signals.

## PKC and Calcium ion Signaling

Increasing evidence indicates that one of the actions exerted by FSH consists in the activation of the protein kinase C (PKC) pathway that is involved in expansion of the cumulus, meiotic maturation of oocytes, and modulation of progesterone production in the ovary (80). Cross-talk between cAMP/PKA and PKC pathways was also described in Sertoli cells (81), where the FSH-dependent activation of these kinases is connected to calcium ion (Ca^2+^) signaling (82), resulting from intracellular release as well as from rapid influx from T-type Ca^2+^ channels (83, 84) or through a Gαh transglutaminase/PLCδ interaction (85). *In vitro* experiments in transiently FSHR over-expressing human embryonic kidney (HEK) and virally transduced human granulosa (KGN) cells demonstrated that intracellular Ca^2+^ increase may occur *via* a molecular mechanism dependent on the interaction between APPL-1 and FSHR, and involving inositol 1,4,5-trisphosphate (IP_3_) (25). Interestingly, IP_3_ production dampens the expression of the aromatase enzyme, at least under FSHR over-expression (86), suggesting an inhibitory role of the APPL-1/IP_3_/Ca^2+^ signaling module on sex steroid synthesis. While further studies are required to confirm these results in the presence of physiological FSHR expression levels, these data show that APPL-1-mediated Ca^2+^ signaling does not necessarily depend on cAMP, as previously demonstrated (87). Moreover, human PKC belongs to a superfamily of about 15 isoenzymes activated upon Gq protein-mediated production of diacylglycerol (DAG) and/or Ca^2+^ by phospholipases at the intracellular level (88). In the mouse ovary, expression of PKC isoforms is dynamic and changes according to the developmental stage, from pre-puberty to the adulthood, suggesting that different isoenzymes may control specific ovarian functions, such as follicular maturation, ovulation, and luteinization (89).

It is known that PKC counteracts the PKA-mediated steroidogenesis through cAMP inhibition in granulosa (90, 91), and this function was further confirmed in both mammalian (92) and avian models (93). Moreover, PKC attenuates the G_α_s protein-dependent signaling (94, 95), as well as proteoglycan synthesis in Sertoli cells (96). Interestingly, several reports demonstrated an up-regulatory role of PKC in Leydig cell steroidogenesis (97). Indeed, the enzyme is involved in the positive modulation of cAMP, pCREB and StAR activation, increasing the rate of steroid synthesis in the mouse Leydig MA-10 cell line (98, 99), and in mouse primary Leydig cells (100). In this case, PKC activation would not depend on FSH, due to the lack of FSHR expression in Leydig cells. On the contrary, PKC up-regulation in ovarian theca cells may be LH-dependent and negatively impacts on androstenedione synthesis *in vitro* (101), suggesting the existence of a sex-specific function of the kinase in regulating the synthesis of sex steroids in androgenic cells.

## The pAKT anti-apoptotic Pathway

FSH binding to its receptor mediates the activation of PI3K, that are enzymes involved in the regulation of cell survival, growth and differentiation (102). In Sertoli cells, FSH increases phosphatase and tensin homolog deleted in chromosome 10 (PTEN) synthesis within minutes, independently of mRNA transcription (79), but rather mediated by FSH-mediated destabilization of several anti-PTEN miRNAs (103). PTEN stabilization in mature rat counteracts PI3K activity, when cell proliferation ceases prior puberty. AKT activation *via* PI3K may occur through both PKA-dependent (104) and independent mechanisms (63), reflecting the relevance of this kinase in modulating proliferative and anti-apoptotic signals in steroidogenic cells. Indeed, in granulosa cells, an interplay between AKT- and cAMP/PKA-pathway up-regulating steroidogenesis was demonstrated (105). Moreover, FSH-dependent activation of the AKT/mammalian target of rapamycin (mTOR) signaling module (106), a positive regulator of cell cycle progression and cell proliferation (107), was also described (108–110). AKT phosphorylation was observed in mouse granulosa cells, where the kinase induces the inactivation of FOXO1 and expression of cyclin D2, resulting in cell proliferation and differentiation in response to FSH (111). In fact, recent genome-wide studies have revealed that most FSH-responsive genes in granulosa cells are FOXO target genes (33). New insights onto FSH-mediated protection from atresia came from the discovery that FOXO nuclear exclusion (inhibition) upon activation of the PIK3/AKT/mTOR signaling pathway prevents granulosa cell autophagy (112, 113). The relevance of pAKT activation for reproduction was highlighted by *in vitro* experiments where mouse preantral follicular granulosa cells were co-cultured with oocytes (114). The presence of granulosa cells inhibited oocyte apoptosis *via* PI3K/AKT, promoting gamete growth. Especially, AKT was described to regulate meiotic resumption in several animal models (115–117). Finally, the AKT pathway is a preferential target of LH (118) and its activation is even enhanced in the presence of FSH (119, 120), suggesting that anti-apoptotic and proliferative stimuli would be required during the late antral follicular phase to prepare the late stages of oocyte maturation and achieve ovulation. Taken together, the PI3K/AKT-pathway may act in concert with mTOR (108) regulating survival signal in the ovary. These signals are fundamental for primordial to Graafian follicles survival, as well as for oocyte maturation and growth. In this context, it is reasonable that the PI3K/AKT anti-apoptotic activity mediated through FSHR is fundamental to counteract cAMP/PKA pro-apototic stimuli and rescue the follicle from atresia (121). In fact, dysregulation of this signaling cascade may impair female gametogenesis and it was described as a cause of infertility (122). Interesting data explaining how signals delivered through the cAMP/PKA- and PI3K/AKT-pathway are counterbalanced come from the analysis of FSH treatment of Sertoli cells. In this model, FSH has a dual, stage-dependent action. While the hormone stimulates the proliferation of immature cells through activation of PI3K/AKT-, mTOR- and ERK1/2-pathways, it preferentially stimulates cAMP production in mature Sertoli cells, resulting in PI3K/AKT inhibition and arrest of cell proliferation (110, 123). While this effect is maybe due to the change of Sertoli cell competence, where PI3K/AKT-pathway activation becomes dependent on paracrine factors during the late stages of the maturation (124), it provides an example of dual regulation of life and death signals exerted by FSH.

## Conclusions

FSH mediates multiple signaling pathways by binding to its unique GPCR (125). At the intracellular level, FSH is capable of promoting cell growth and survival opposed to steroidogenic signals cross-linked to apoptosis, resulting in a fine-tuned regulation of the gametogenesis and, in general, of reproduction. In the male gonads, FSH induces proliferation of Sertoli cells *via* AKT- and ERK1/2-pathways and the role of these signaling cascades, which are proliferative and anti-apoptotic, is reflected during folliculogenesis, oocyte maturation, and growth in the ovary. The synthesis of steroid hormones mainly mediated by cAMP/PKA-pathway activation is a primary endpoint in FSH functioning in the granulosa cell during the antral stage of folliculogenesis. Estrogens are the final products required for proper development of the dominant follicle, at the cost of scarifying others which become *atretic*. It is well known that follicular *atresia* is due to lowering of FSH support. However, *in vitro* data support unexpected, stage-specific pro-apoptotic signals delivered by the hormone that may play a role *in vivo* and this issue merits further investigations.

## Author Contributions

All authors listed have made a substantial, direct and intellectual contribution to the work, and approved it for publication.

### Conflict of Interest Statement

The authors declare that the research was conducted in the absence of any commercial or financial relationships that could be construed as a potential conflict of interest.
